# Optimization of Millet Malting Parameters Using Artificial Neural Network and Response Surface Methodology

**DOI:** 10.1002/fsn3.70214

**Published:** 2025-04-30

**Authors:** Fatemeh Erfaniannejad Hosseini Nabadou, Masoumeh Moghimi, Aminallah Tahmasebi, Hamid Bakhshabadi

**Affiliations:** ^1^ Department of Food Science and Technology, Gonbad Kavoos Branch Islamic Azad University Gonbad Kavoos Iran; ^2^ Department of Chemistry, Gonbad Kavoos Branch Islamic Azad University Gonbad Kavoos Iran; ^3^ Department of Agriculture, Minab Higher Education Center University of Hormozgan Bandar Abbas Iran

**Keywords:** artificial neural network, germination period, malting process, millet, steeping duration

## Abstract

The quality of malt produced from cereals is significantly influenced by various factors, including steeping and germination periods. Monitoring these factors and their effects on malt grain characteristics is often time‐consuming and costly. In this context, this study aimed to predict trends in changes to certain characteristics of millet‐derived malt, influenced by varying steeping durations (24–48 h) and germination times (5–9 days). Changes in these characteristics were predicted using response surface methodology (RSM), which incorporated a central composite design and an artificial neural network (ANN). The findings indicated that increasing the steeping and germination durations led to a decrease in malting efficiency, thousand grain weight, and true density of the samples. Conversely, the cold‐water extract efficiency, the Kolbach index, and the extract color increased. The optimization process revealed that to achieve the highest‐quality malt, the steeping duration should be 42.54 h, followed by a germination period of 5 days. Under these conditions, the malting efficiency reached 75.44%, with a thousand grain weight of 4.85 g, a true density of 977.43 kg/m^3^, a cold‐water extract efficiency of 9.19%, a Kolbach index of 32.45%, and an extract color value of 13.87. An analysis of different neural networks revealed that the feed‐forward backpropagation network with a 2‐6‐6 topology was the best‐performing model. This network achieved a correlation coefficient greater than 0.999 and a mean squared error of less than 0.00001. It employed the hyperbolic tangent sigmoid transfer function, the resilient backpropagation learning algorithm, and 1000 learning cycles. Furthermore, a comparison of the correlation coefficients derived from the RSM and the ANN demonstrated that the ANN method is superior for predicting changing trends in millet grains during the malting process.

## Graphical abstract

With increasing steeping and germination time, the malting efficiency, thousand‐grain weight, and true density of millet malt decrease, while the cold water extract efficiency increases. The results showed that to achieve the highest quality malt, steeping and germination times should be 42.54 h and 5 days, respectively. The artificial neural network method is more effective for predicting the trend of changes in the characteristics of millet malt grains during the malting process compared to the response surface method.

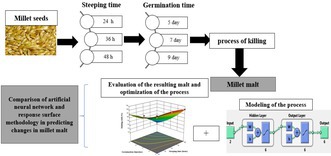

## 1 Introduction

The development and establishment of human civilizations are profoundly connected to the cultivation of grains (Calvi et al. 2023). Grains are a fundamental component of staple foods worldwide, and their fermentation is a traditional technique for producing desirable food products. For thousands of years, grain‐based foods have been a primary source of nutrition, particularly in developing and densely populated regions, where they are consumed raw, cooked, or fermented (Bouakkaz et al. 2024; Calvi et al. 2023). The malting process is a sophisticated biotechnological method that involves several stages, including steeping, germination, and drying of cereal grains under specific temperature and humidity conditions. The primary aim of malting is to produce hydrolytic enzymes and to degrade the cell walls, proteins, and starches found in the endosperm, thereby enhancing the crispness and brittleness of the grains (Ghodsvali, Mokhtarian, et al. 2013). In the malting industry, a variety of grains, such as barley, wheat, sorghum, millet, and triticale, are utilized. However, barley is favored over other grains due to its distinct chemical composition, beneficial changes during germination, and the protective function of its husk during transport (Bakhshabadi et al. 2012). Millet ( *Panicum miliaceum* L.) is ranked as the sixth most significant cereal grain globally. It is widely utilized in many developing countries for food products, animal feed, and as a raw material for the production of low‐viscosity foods, baked goods, malt, starch, sugar, syrup, and ethanol. This vital grain is also known for its heat and drought resistance, making it well‐suited to withstand the adverse effects of projected climate change. These advantages, combined with its exceptional nutritional profile, low glycemic index, high phytochemical content, and suitability for individuals with gluten sensitivities, have fueled growing interest in this ancient grain in recent years. Millet has recently garnered considerable attention, particularly following the United Nations General Assembly's declaration of 2023 as the International Year of Millets. This grain has the potential to thrive in challenging conditions with minimal resources, effectively addressing food security and nutritional needs (Adu et al. 2024; Elavarasan et al. 2024). Various factors influence the quality of malt derived from grains, including the variety and type of seed, steeping and germination periods, drying conditions, and extraction methods (Bakhshabadi 2011). In this context, several researchers have investigated the effects of steeping and germination duration on cereal malt. For instance, Ghodsvali, Bakhshabadi, et al. (2013) and Ghodsvali, Mokhtarian, et al. (2013) demonstrated that an increase in steeping and germination time enhances the extraction efficiency of barley malt (Ghodsvali, Bakhshabadi, et al. 2013). In another study, Bakhshabadi et al. (2015) demonstrated that an increase in steeping and germination duration leads to a decrease in malting efficiency (Bakhshabadi et al. 2015). Furthermore, Eftekhari Yazdi et al. (2023) found that increasing steeping time enhances the extraction efficiency of quinoa (Eftekhari Yazdi et al. 2023). However, there is limited information addressing the effects of these conditions on millet malt, particularly given its unique characteristics and the reliance of many countries on imported barley for malting. Additionally, artificial neural network (ANN) is a computational model inspired by the functioning of biological neural networks in the human brain. The ability of artificial neural networks to identify nonlinear relationships makes them particularly useful in situations where the interactions among process variables are complex. Artificial neural networks can learn from data and identify patterns and trends that may be challenging for conventional methods to detect. These networks can be updated continuously and improved with the availability of additional data. By leveraging the computational capabilities of artificial neural networks, researchers can investigate a range of scenarios and predict outcomes, overcoming the limitations of relying solely on physical experiments. This approach expedites the decision‐making process and reduces the necessity for large‐scale experiments (Singer et al. 2024). Numerous studies have effectively utilized artificial neural networks to predict the properties of malt produced from grains (Ajayi et al. 2023; Ghodsvali et al. 2016; Ghodsvali, Mokhtarian, et al. 2013). Traditionally, optimizing chemical processes requires analyzing the influence of one variable at a time on a test response, while maintaining other variables at a constant level. This method, known as one‐variable‐at‐a‐time optimization, has a significant limitation as it fails to account for the interactive effects among the studied variables. Another disadvantage of single‐variable optimization is the need for a greater number of experiments in the research process, which increases time, costs, and the consumption of reagents and materials. To address this challenge, analytical methods have been optimized using multivariate statistical techniques. One of the most relevant multivariate techniques applied in analytical optimization is response surface methodology (RSM). RSM is a set of mathematical and statistical techniques based on fitting a polynomial equation to experimental data (Bezerra et al. 2008). Jensen (2017) emphasized the importance of RSM in optimizing products and processes through designed experiments. The study emphasized the efficiency of RSM in reducing the number of experiments needed for optimization, leading to cost savings and quicker results. Given the scarcity of studies evaluating the comparative effectiveness of RSM and artificial neural networks in predicting malt characteristics under diverse steeping and germination conditions, as well as the high costs and time associated with laboratory analyses of malt quality factors, this research aims to optimize the millet malt production process using RSM. Additionally, the study aims to compare the models derived from this method under optimal conditions with those from artificial neural networks in predicting the characteristics of malt obtained from millet under varying steeping and germination times.

## 2 Materials and Methods

### 2.1 Preparation of Millet Grains and Conditions for the Production of Malt

To conduct this research, millet grains of the Morvarid cultivar were obtained from the Gonbad Agricultural Research Center. The seeds were cleaned using a sieve and then manually washed. After cleaning, the seeds were weighed and divided into three equal portions, each subjected to steeping to facilitate various tests, such as measurements of protein, moisture, and fiber content. To achieve the necessary water absorption, the millet grains were immersed in containers filled with a specific volume of water, maintained at approximately 20°C and a hardness of around 250 ppm, for 24, 36, and 48 h. After steeping, the excess water was removed from the millet grains through rinsing. The seeds were then placed in a germinator (Tabai Espec Corp, Japan) at a temperature of 17°C–20°C to complete the germination process for 5, 7, and 9 days. Following germination, the samples were dried in an oven at temperatures of 55°C–65°C for 48 h. The rootlets were then separated manually and through sieving. In the next phase, the dried malts were ground into a fine powder using a grinder, and the extraction process was carried out. The soaked malt was separated from the remaining malt using Whatman filter paper grade No. 1 and a vacuum pump (Bakhshabadi et al. 2015; Ghodsvali et al. 2016). The tests conducted on the malt grains and the produced extract are as follows:

### 2.2 Malting Efficiency

Malting efficiency was determined using a digital scale that offers an accuracy of 0.01 g, applying the following Equation (1) (Ghodsvali et al. 2016):

(1) $$\text{Malting yield}=\frac{\text{Weight of malt grains}}{\text{Weight of millet grains}}\times 100$$

### 2.3 Thousand Grain Weight

A total of one thousand seeds were randomly selected and weighed to determine their weight, with the results reported in grams (Bakhshabadi et al. 2015).

### 2.4 The True Density

The true density of the malt samples ($\rho_{t}$) was measured using a pycnometer, following the fluid displacement principle with toluene. Specifically, the true density of the samples was calculated from the ratio of the weight of 10 measured grains (*m*_*t*_) at 20°C to the volume of the pycnometer (*v*) (Bakhshabadi et al. 2015).

(2) $$\rho_{t}=\frac{m_{t}}{v}$$

### 2.5 Efficiency of Cold‐Water Extract

A total of 500 mL of distilled water was added to twenty‐five grams of finely ground malt at 20°C. The mixture was stirred at 20‐min intervals and filtered using a vacuum pump (Eliminator JB, USA). The efficiency of the cold‐water extract was assessed by measuring the specific gravity of the liquid obtained from straining the soaked malt using the cold extraction method. This measurement was conducted using a pycnometer, and the corresponding Brix value was referenced from the Plato Brix table (Ghodsvali, Bakhshabadi, et al. 2013). The yield percentage of the cold‐water extract was then calculated using the following equation:

(3) $$E=\frac{\left(800+M\right)P}{100-P}$$

In this equation, *E* represents the percentage of extract obtained through cold water, *M* denotes the percentage of malt moisture utilized in the experiment, and *P* indicates the Brix value of the extract according to the Plato table.

### 2.6 Ratio of Soluble Nitrogen to Total Nitrogen (Kolbach Index)

Following the assessment of the total nitrogen in the malt and the soluble nitrogen in the total extract, the nitrogen percentage was calculated using the following Equation (4) via an automatic macro Kjeldahl device (Auto Analyser 130 Tecator CO, Denmark) (Eftekhari Yazdi et al. 2023).

(4) $$\text{Kolbach index}=\frac{\text{Soluble nitrogen}}{\text{Total nitrogen of malt}}\times 100$$

### 2.7 Extract Color

In accordance with the AOAC method (2008), the extract was prepared using the temperature‐timing technique. Next, 5 g of silit was added to 100 mL of the prepared extract to facilitate clarification, and the mixture was incubated for 5 min. The solution was then filtered using Whatman filter paper grade No. 1. The color of the extract was determined using the following equation:

(5) $$\text{Extract color}=10\times A_{430}$$

*A*_430_ represents the absorption value of the extract measured with a spectrophotometer (Biochrom, UK) at a wavelength of 430 nm (AOAC 2008).

### 2.8 Statistical Analysis and Modeling of the Malting Process

To assess the fixed parameters of the study, steeping time (24–48 h) and germination time (5–9 days) were utilized in a central composite design to evaluate their effects on malting efficiency, thousand grain weight, true density, cold‐water extract efficiency, Kolbach index, and extract color. Thirteen experiments with five replications at the central point (steeping and germination times of 36 h and 7 days, respectively) were conducted. This design allowed for the estimation of all coefficients of the quadratic regression model and the interaction between factors. The primary focus of this study was to explore the interaction between factors and determine the optimal conditions for millet malt production, hence the selection of a response surface statistical design. To analyze the behavior of the response surfaces, a quadratic polynomial equation was applied to each independent variable. The quality and accuracy of the regression model, as well as the goodness of fit, were assessed using model analysis parameters, lack of fit, and the coefficient of determination. Statistical analysis was performed using Design Expert version 12 software. To determine the optimal neural network, the neural network tool in MATLAB (R 2013a) software was used. In designing this network, steeping and germination times were defined as two inputs in a two‐row matrix, while malt extraction yield, thousand grain weight, true density, cold‐water extract efficiency, Kolbach index, and extract color were defined as targets in a six‐row matrix. Various neural networks, including different activation functions, learning rates, and varying numbers of neurons, were designed in the hidden layer, and their performance was assessed using two evaluation criteria: the coefficient of determination (*R* ^2^) and mean squared error (MSE), as respectively defined by Equations (6) and (7). Initially, by testing different neural networks, the feedforward neural network with the highest performance was selected, and the number of learning cycles was set to 1000. Considering these factors, various neural networks containing a hidden layer with a varying number of neurons, ranging from 1 to 10, were designed. To connect the input layer to the hidden layer, hyperbolic tangent sigmoid, logarithmic, and linear activation functions were employed during various stages of the network's trial‐and‐error process. Additionally, a constant linear activation function was used for the connection between the hidden layer and the output layer. Furthermore, two distinct learning patterns, namely the Levenberg–Marquardt learning algorithm and resilient backpropagation, were applied across different networks, and their impact on the accuracy of the networks was evaluated. In these equations, $Y_{\mathrm{pi}}$ represents the ratio of features predicted by the network, $Y_{\mathrm{ei}}$ shows the proportion of the experimental features, and N indicates the total number of observations.

(6) $$R^{2}=1-\frac{\sum_{i=1}^{N}{\left(Y_{\mathrm{pi}}-Y_{\mathrm{ei}}\right)}^{2}}{\sum_{i=1}^{N}{\left(Y_{\mathrm{pi}}-\bar{Y}\right)}^{2}}$$

(7) $$\mathrm{MSE}=\frac{1}{N}\sum_{i=1}^{N}{\left(Y_{\mathrm{pi}}-Y_{\mathrm{ei}}\right)}^{2}$$

Using raw input data can decrease the efficiency and accuracy of the network. Therefore, it is crucial to normalize the input data before integrating it into the network. If the normalization step is omitted, the network may not achieve convergence during the training phase, leading to inadequate results. In this study, Equation (8) was used to calibrate the data, ensuring that the inputs and outputs are standardized within the range of 0–1.

(8) $$V_{N}=\frac{V_{R}-V_{\min}}{V_{\max}-V_{\min}}$$

In this equation, *V*_R_ represents the raw input data, *V*_N_ shows the normalized data, while *V*_max_ and *V*_min_ indicate the maximum and minimum values of the initial data, respectively (Dolatabadi et al. 2016; Farzaneh et al. 2018). In the present study, the dataset was partitioned such that 60% (26 points) was allocated for training, 15% for validation, and the remaining portion (10 points) was designated for testing the network. A total of 43 samples were analyzed by the neural network.

### 2.9 Analysis of the Performance of RSM and ANN for the Prediction of the Measured Characteristics

For this purpose, the coefficient of determination (*R* ^2^) of the models was employed, revealing that equations with higher *R* ^2^ values were indicative of more efficient performance in predicting the characteristics of millet malt.

## 3 Results and Discussion

### 3.1 The Effect of the Steeping and Germination Time on the Performance of the Malting Process

**FIGURE 1:**
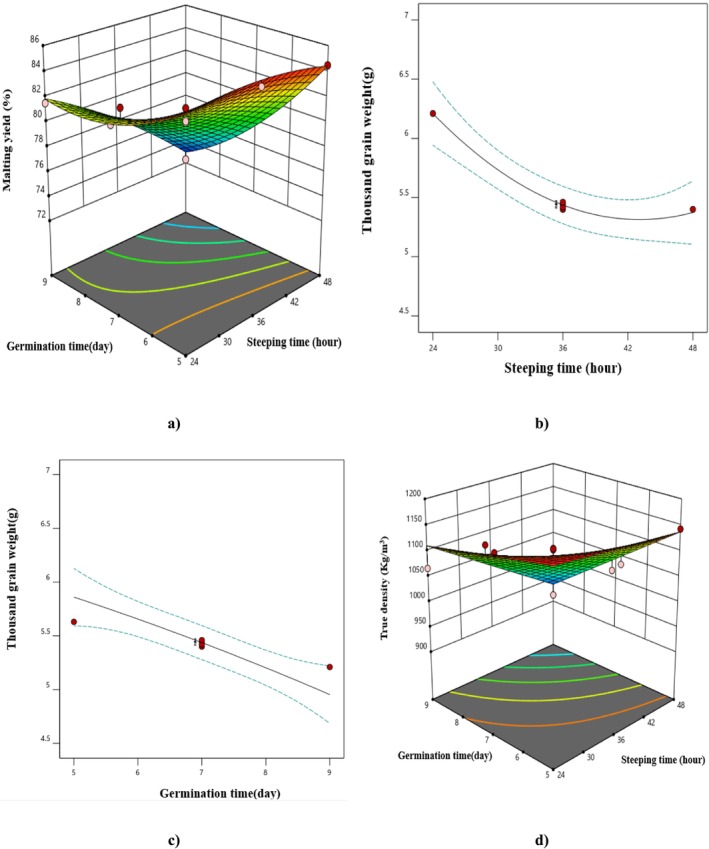
The effect of (a) steeping and germination time on malting yield, (b) steeping time on thousand grain weight, (c) germination time on thousand grain weight and (d) steeping and germination time on true density.

The analysis of variance for the data obtained from the performance of the malting process (Table 1) indicated that all parameters, except for the second‐degree parameter of steeping time, were significant in relation to malting efficiency (*p* < 0.05). Figure 1a illustrates that an increase in steeping and germination times results in a decrease in malting efficiency, with the linear effect of germination time being more pronounced than that of other parameters, as evidenced by the *F* values and coefficients presented in Table 2. The increase in steeping and germination times led to increased cellular respiration, greater solute transfer from the grains into the water, and nutrient consumption by the grains for plant growth, all contributing to the decrease in malting efficiency (Eftekhari Yazdi et al. 2023). The loss associated with the steeping process is primarily due to the removal of dust, the dissolution of grain materials through solid loss, and the metabolic activity of the grains, resulting in the release of CO_2_ and a small amount of ethanol. The reduction in weight during the steeping phase of barley has been reported to be between 0.5% and 1.5% (Briggs 1998). Eneje et al. (2004) also showed that an increase in steeping time led to a decrease in malting efficiency for corn grains, which aligns with the findings of our study (Eneje et al. 2004). Other studies have reported that the decrease in malting efficiency occurs due to metabolic activity and the separation of rootlets, with longer periods of germination and steeping contributing to a greater decrease in malting efficiency (Bakhshabadi et al. 2015). Some studies have reported that an increase in germination duration correlates with the degradation of beta‐glucans and other carbohydrates, thereby affecting malting efficiency (Turner et al. 2019).

**TABLE 1:** Analysis of variance for determined parameters.

Source	Malting yield		*p*	Thousand grain weight		*p*	True density		*p*	Cold‐water extract		*p*	Kolbach index		*p*	Extract color		*p*
	Sum of squares	*F*		Sum of squares	*F*		Sum of squares	*F*		Sum of squares	*F*		Sum of squares	*F*		Sum of squares	*F*	
Model	133.97**	69.95	< 0.0001	2.67**	20.57	0.005	44395.67**	19.89	0.0003	3.09**	9.44	0.005	75.61**	43.94	< 0.0001	20.93**	33.71	< 0.0001
X_1_	32.99**	86.14	< 0.0001	1.05**	40.51	0.0004	15000.00**	20.16	0.0015	1.73**	10.54	0.009	8.17**	27.73	0.0018	14.26**	114.84	< 0.0001
X_2_	78.26**	204.33	< 0.0001	1.23**	47.57	0.0002	24066.67**	32.35	0.0003	1.37*	8.33	0.016	57.97**	168.45	< 0.0001	4.59**	36.99	0.0005
X_1_X_2_	17.68**	46.16	0.0003	0.00 ns	0.001	0.9522	5329.00*	7.16	0.0254	—	—	—	0.11 ns	0.31	0.598	0.19 ns	1.45	0.267
X_1_^2^	1.01 ns	2.63	0.148	0.35**	13.40	0.0081	—	—	—	—	—	—	1.21 ns	3.52	0.103	1.75**	14.13	0.0071
X_2_^2^	5.00s**	13.06	0.0086	0.00 ns	0.09	0.763	—	—	—	—	—	—	4.94**	14.35	0.0068	0.02 ns	0.19	0.6744
Residual	2.68	—	—	0.18	—	—	6695.26	—	—	1.64	—	—	2.41	—	—	0.87	—	—
Lack of fit	2.58	34.42	—	0.18	104.77	—	6690.06	1029.24	—	1.63	145.42	—	2.36	60.44	—	0.86	113.19	—
Pure error	0.10	—	—	0.00	—	—	5.20	—	—	0.01	—	—	0.05	—	—	0.01	—	—
Cor total	136.65	—	—	2.85	—	—	51090.92	—	—	4.73	—	—	78.02	—	—	21.80	—	—

Abbreviations: X_1_, steeping time; X_2_, germination time.

* and **Significance at 1% and 5% level, and ns reported as non‐significant.

**TABLE 2:** Predictive models of dependent variables.

Number	Dependent variable	Equation	*R* ^2^	*R* ^2^‐adj	CV
1	Malting yield	*y* = +80.32 − 2.35 X_1_ − 3.61 X_2_ − 2.10 X_1_X_2_ − 0.60 X_1_^2^ + 1.35 X_2_^2^	0.980	0.966	0.767
2	Thousand grain weight	*y* = +5.44 − 0.42 X_1_ − 0.45 X_2_ + 0.005 X_1_X_2_ + 0.36 X_1_^2^ − 0.03 X_2_^2^	0.936	0.890	2.88
3	True density	*y* = +1087.92 − 50.00 X_1_ − 63.33 X_2_ − 36.50 X_1_X_2_	0.869	0.825	2.51
4	Cold‐water extract	*y* = +8.42 − 0.54 X_1_ − 0.48 X_2_	0.753	0.684	4.81
5	Kolbach index	*y* = +30.15 + 1.17 X_1_ + 3.11 X_2_+ 0.16 X_1_X_2_ − 0.66 X_1_^2^ − 1.34 X_2_^2^	0.969	0.947	2.01
6	Extract color	*y* = +11.90 + 1.54 X_1_+ 0.88 X_2_ + 0.21 X_1_X_2_ + 0.79 X_1_^2^ − 0.09 X_2_^2^	0.960	0.932	2.88

### 3.2 The Effect of Various Parameters on the Thousand Grain Weight

The findings presented in Table 1 indicate that the linear parameters of steeping and germination times, as well as the quadratic parameter of steeping time, significantly influenced the thousand grain weight in the samples (*p* < 0.05). Consequently, linear graphs were utilized for drawing this characteristic due to the non‐significant interaction effects of these parameters. Figure 1b,c shows that an increase in both steeping and germination times resulted in a decrease in the thousand grain weight of the produced malt. Furthermore, based on the *F* values in Table 1 and the coefficients from Table 2, it can be concluded that the most substantial effect on the thousand grain weight of malt was related to the linear parameter of germination time. The reduction in the thousand grain weight can be attributed to the physiological activities of the grains. As the duration of germination increases, the physiological activity of the seeds also rises, leading to enhanced enzyme activity and greater utilization of food reserves for energy and growth, which ultimately results in a decrease in seed compounds during malting. Starch serves as a carbohydrate source and is considered a key energy provider for grains during germination. The decrease in the thousand grain weight can be linked to a reduction in the starch content of the seeds (Makhaye et al. 2021; Tabatabaei 2014). Furthermore, an increase in steeping duration leads to a higher release of substances from the grains into the water, which subsequently reduces the thousand grain weight of malt (Bakhshabadi et al. 2015).

### 3.3 The True Density

The selection of the 2FI model by the software indicated that only the linear parameters and the interaction effect of steeping and germination times had a significant effect on the true density of malt grains at the 5% level (Table 1). The results demonstrated that an increase in steeping and germination times led to a reduction in the density of the samples (Figure 1d). This decrease in density can be explained by a decrease in grain weight due to increased physiological activity of the grains and an increase in grain volume during the malting process (Bakhshabadi et al. 2015). Some studies have linked the reduction in malt grain density to the breakdown and degradation of complex cellular compounds, such as proteins and starch, which result in decreased interparticle gravitational interaction (Murungweni et al. 2024). Some studies have also attributed the increase in grain volume, and consequently the decrease in density, to the increase in both the width and thickness characteristics of the malt grains compared to the initial grains (Maghsoudlou and Kashiri 2012). Ghodsvali et al. (2016) and Bakhshabadi et al. (2012) similarly reported that the density of malt produced from barley grains decreases with increased steeping and germination times. The most significant effect on the true density of the samples was attributed to the linear parameter of germination time, followed by the linear parameter of steeping time (Table 2).

### 3.4 The Efficiency of Cold‐Water Extract

**FIGURE 2:**
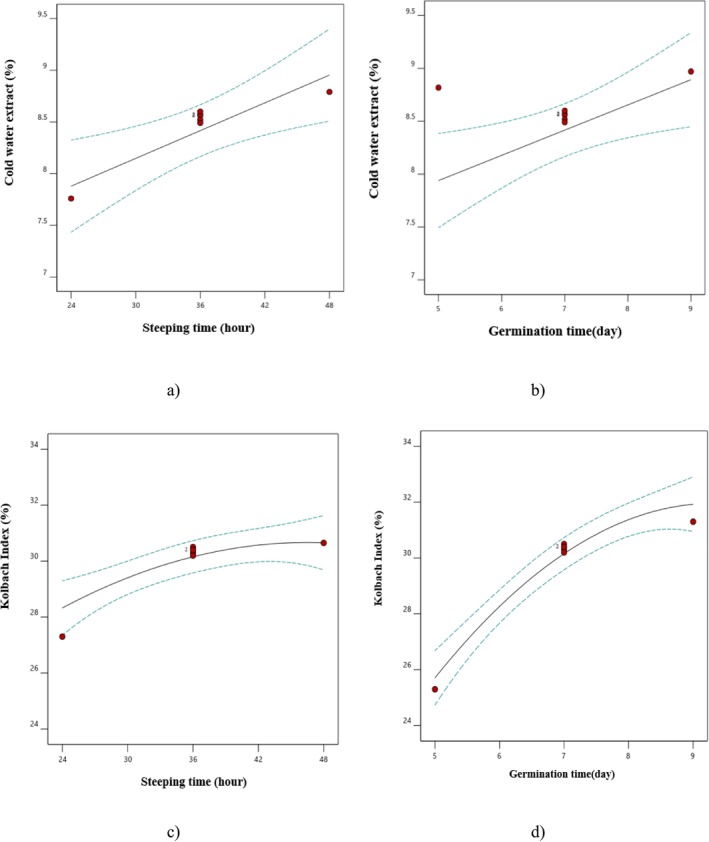
The effect of (a) steeping time on cold‐water extract, (b) germination time on cold‐water extract, (c) steeping time on kolbach index and (d) germination time on kolbach index.

Table 1 shows that the linear model was identified as the most suitable for fitting the data regarding the effects of steeping and germination times on the efficiency of cold‐water extract, with only linear parameters being statistically significant (*p* < 0.05). Furthermore, the results presented in Table 2 and the *F*‐values suggested a more pronounced effect of the linear parameter of steeping time on the efficiency of cold‐water extract. The findings also showed that extending the steeping and germination time (Figure 2a,b) led to an increase in the efficiency of cold‐water extract. The efficiency of cold‐water extract is a critical criterion for assessing malt quality. It inhibits enzyme activity under mildly alkaline conditions at 20°C, indicating compounds solubilized during the germination phase (Palmer 1989). Briggs (1998) reported a direct correlation between the efficiency of cold‐water extract and the degree of grain modification during the germination process. Briggs also noted that optimal endosperm modification is typically associated with an increase in the concentration of soluble compounds of malt, which results in higher efficiency of cold‐water extract and subsequently enhances aroma and color during the drying process (Briggs 1998). Another reason for the increased efficiency of cold‐water extraction with increased steeping and germination periods is the enhanced activity of enzymes, breakdown of proteins, and other complex compounds in the seed tissue and the release of these compounds into the extract, thereby increasing both the Brix value and the extraction efficiency (Miano et al. 2015; Ofoedu et al. 2021). Farzaneh et al. (2017) also demonstrated that increasing the germination period results in a greater degradation of starch and protein, which is linked to increased enzymatic activity (Farzaneh et al. 2017). This naturally facilitates the release of these compounds into the extract, further enhancing the efficiency of cold‐water extraction. Other studies have similarly reported an increase in steeping and germination times contributes to greater extraction efficiency (Ghodsvali, Bakhshabadi, et al. 2013).

### 3.5 The Effect of Various Studied Parameters on the Kolbach Index

One of the most significant parameters in the malting process is the Kolbach index, which indicates the extent of degradation or breakdown of the grain proteins. This index has a direct correlation with extraction efficiency and soluble protein content (Calvi et al. 2023; Jin et al. 2014). According to the analysis of variance (Table 1), it was determined that the interaction effects of steeping and germination times, as well as the quadratic effect of steeping time, did not significantly influence the Kolbach index (*p* > 0.05). Furthermore, based on the *F* values and coefficients from Table 2, it can be concluded that the linear parameter of germination time had the most substantial effect on the Kolbach index of the samples. Additionally, an increase in both steeping and germination times resulted in a higher Kolbach index (Figure 2c,d). This increase can be attributed to a greater amount of soluble protein in the extract (Calvi et al. 2023). Some studies have attributed the increase in this index to enhanced enzyme activity during germination (Jin et al. 2014). In alignment with these findings, Ghodsvali, Bakhshabadi, et al. (2013) and Ghodsvali, Mokhtarian, et al. (2013) showed that the Kolbach index of the samples was increased with the higher germination time, which was associated with improvements in extract and favorable changes in the grain endosperm during the germination (Ghodsvali, Bakhshabadi, et al. 2013). Eftekhari Yazdi et al. (2023) also reported that extending the steeping time resulted in an increase in the Kolbach index of samples, which is in agreement with the findings of this study.

### 3.6 Extract Color

**FIGURE 3:**
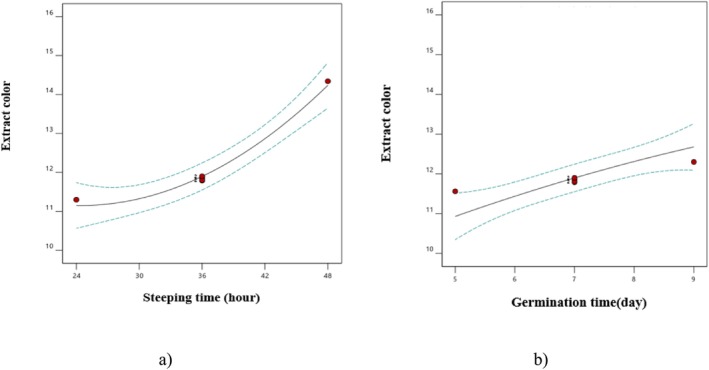
The effect of (a) steeping time on extract color and (b) germination time on extract color.

The analysis of variance regarding the effects of steeping and germination times revealed that all parameters, except for the interaction effect between steeping and germination times, as well as the quadratic parameter of germination time, were significant at the 5% level (Table 1). Additionally, the *F* values and Table 2 showed that the linear parameter of steeping time had the most substantial effect on the extract color. The results demonstrated that an increase in both steeping and germination times led to a rise in the color of the extracts (Figure 3a,b). The primary factor contributing to the formation of extract color is the non‐enzymatic Maillard reactions that occur during the drying phase of green malt. Given that the objective of producing pale malt is to achieve maximum yield of hot water extract, it is desirable to increase the levels of reducing sugars and amino acids. Therefore, to minimize the rate of Maillard reactions during the drying process, air circulation is essential to prevent the formation of melanoidin pigments despite the presence of color‐forming precursors (Francis 1999). The process of gradual extraction at elevated temperatures results in the formation and accumulation of levels of soluble sugars and amino acids, which enhance the development of extract color during the malting process (Briggs 1998). Other studies have also highlighted that the color of the extract enhances with increased steeping and germination time during the malting process, which is in agreement with the findings of our study (Eftekhari Yazdi et al. 2023; Ghodsvali, Bakhshabadi, et al. 2013).

### 3.7 Optimization of the Production Process of Millet Malt

In order to determine the optimal conditions for producing millet malt, a steeping duration ranging from 24 to 48 h and a germination period between 5 and 9 days were evaluated. The malting process of millet grains was optimized under these conditions to achieve maximum malting efficiency, enzyme, Kolbach index, and optimal cold‐water extract yield. The results indicated that the steeping and germination durations should be optimized at 42.54 h and 9 days, respectively, to achieve the desired results. Under these optimal conditions, a desirability score of 0.820 was achieved. It is noteworthy that the measured characteristics under the optimal conditions included malting efficiency of 75.44%, thousand grain weight of 4.85 g, the true density of 977.43 kg/m^3^, cold‐water extract efficiency of 9.19%, Kolbach index of 32.45%, and extract color of 13.87.

### 3.8 Modeling the Malting Process Using Artificial Neural Networks

**FIGURE 4:**
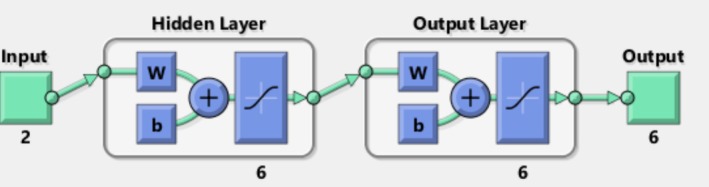
The schema of a selected optimized network containing 2 neurons in input layer, 6 neurons in hidden layer with activation function of hyperbolic sigmoid tangent, and 6 neurons in hidden layer with hyperbolic sigmoid tangent activation function.

Table 3 shows the comparison of the effect of the number of neurons in the hidden layer and the type of learning pattern on the prediction accuracy of feedforward neural networks utilizing hyperbolic tangent sigmoid, logarithmic, and linear activation functions, with a number of learning cycles of 1000. Based on the mean squared error values and correlation coefficients presented in these tables, the feedforward neural network employing the hyperbolic tangent activation function, resilient backpropagation learning algorithm, and a topology of 2‐6‐6 (where the input layer consists of 2 neurons, a hidden layer contains 6 neurons, and the output layer has 6 neurons, as depicted in Figure 4) was selected as the optimal neural network, achieving a correlation coefficient exceeding 0.999 and a mean squared error of 0.00001.

**TABLE 3:** Comparison of the effect of the number of neurons in the hidden layer and the type of learning function and activation function on predicting the accuracy of various properties.

Neurons number	Hyperbolic sigmoid tangent				Sigmoid logarithm				Linear			
	Trainlm		Trainrp		Trainlm		Trainrp		Trainlm		Trainrp	
	*R* ^2^	MSE	*R* ^2^	MSE	*R* ^2^	MSE	*R* ^2^	MSE	*R* ^2^	MSE	*R* ^2^	MSE
2	0.958	0.00587	0.777	0.07671	0.972	0.00659	0. 955	0. 01123	0.963	0.00356	0.956	0.01929
3	0.952	0.00741	0.921	0.026001	0.978	0.00048	0.891	0.02305	0.975	0.00416	0.970	0.00416
4	0.998	0.00009	0.997	0.001013	0.772	0.01359	0.990	0.00022	0.983	0.00391	0.965	0.00124
5	0.791	0.02342	0.998	0.00007	0.966	0.00246	0.996	0.00010	0.942	0.00897	0.979	0.00271
6	0.998	0.00008	**0.999**	**0.00001**	0.996	0.00012	0.997	0.00103	0.965	0.00156	0.943	0.00561
7	0.962	0.00495	0.998	0.00004	0.988	0.05104	0.993	0.00204	0.993	0.00015	0.803	0.01986
8	0.999	0.00052	0.991	0.00070	0.997	0.00007	0.997	0.00009	0.989	0.00021	0.969	0.00356
9	0.943	0.00843	0.906	0.016255	0.998	0.00004	0.998	0.00005	0.987	0.00042	0.977	0.00216
10	0.949	0.00798	0.813	0.01855	0.998	0.00010	0.991	0.00031	0.991	0.00032	0.995	0.00199

*Note:* The bold values show the best‐performing number of neurons in the hidden layer and the type of learning function and activation function on predicting the accuracy of various properties.

The artificial neural network is developed by applying training algorithms. As the data goes through all stages of training, testing, and validation, the correlation coefficient between the experimental and predicted data increases, and the error rate decreases compared to other modeling methods (Karami et al. 2022). Another reason for the high correlation coefficient and low mean square error is data normalization and proper configuration of the neural network. This leads to a reduction in the mean square error (Gomaa et al. 2022; Karami et al. 2022). Expressing the parameters considered in the database in a non‐dimensional form is of particular significance for the calibration and overall function of the ANN, since this improves its ability to provide accurate predictions. To avoid problems associated with low learning rates of the ANN, it is more effective to further normalize the values of the parameters between appropriate upper and lower limiting values (Ahmad et al. 2018).

Considering the selected neural network topology, which is structured as 2‐6‐6, the weight matrix for the connection from the input layer to the hidden layer will be a 6 × 2 matrix (linking 2 neurons from the input layer to 6 neurons in the hidden layer). Similarly, the weight matrix for the connection from the hidden layer to the output layer will be a 6 × 6 matrix (connecting 6 neurons from the hidden layer to 6 neurons in the output layer). These matrices will be referred to as matrices A and B, respectively.

$$A=\left(\begin{array}{cc} -5.988 & -1.946\\ -1.884 & -4.584\\ -4.636 & 1.830\\ 5.117 & 3.077\\ -7.724 & 3.643\\ 2.916 & -0.041 \end{array}\right)$$

$$B=\left(\begin{array}{cccccc} 1.937 & 0.102 & -0.525 & -2.065 & 0.607 & 1.882\\ 2.955 & -1.671 & -1.165 & -5.463 & 0.529 & 1.785\\ 5.632 & -2.321 & -1.654 & -7.378 & -0.823 & -0.364\\ -3.799 & 1.851 & 1.365 & 4.670 & -1.152 & -0.952\\ -2.869 & 0.822 & 1.089 & 5.286 & 0.006 & -0.528\\ -1.934 & 0.301 & -0.005 & 1.659 & 0.057 & 0.834 \end{array}\right)$$

Additionally, the bias matrices for the hidden layer (matrix C) and the output layer (matrix D) will be 6 × 1.

$$C=\left(\begin{array}{c} 6.494\\ -2.648\\ 4.266\\ 1.219\\ -4.321\\ 4.286 \end{array}\right)$$

$$D=\left(\begin{array}{c} -1.887\\ 1.132\\ -0.377\\ -0.377\\ -1.132\\ 1.887 \end{array}\right)$$

A numerical neuron is typically made up of *n* inputs *X*_*j*_, where *j* represents the source neuron ranging from 1 to *n*. Each input *X*_*j*_ is weighted before entering the main processor core by being multiplied by a coefficient (*W*_*j*_). This operation simulates the effect of the synapse on the input signal before reaching the desired neuron. Additionally, each neuron has a bias (*W*_0_). The bias is an input signal to neuron *j* from neuron O, with the output always being one (*X*_0_ = 1) and the weight between neuron O and neuron *j* always being *W*_0_. A standard neuron also includes a threshold, and if the net input to the neuron is greater than or equal to this threshold, the neuron fires, mirroring the threshold value in a typical neuron (Ebrahimi et al. 2024). On the other hand, having the weight and bias values allows us to obtain the relationships created by the neural model and confirm the number of input and output neurons in the optimal neural network (Farzaneh et al. 2018; Bakhshabadi et al. 2017).

**FIGURE 5:**
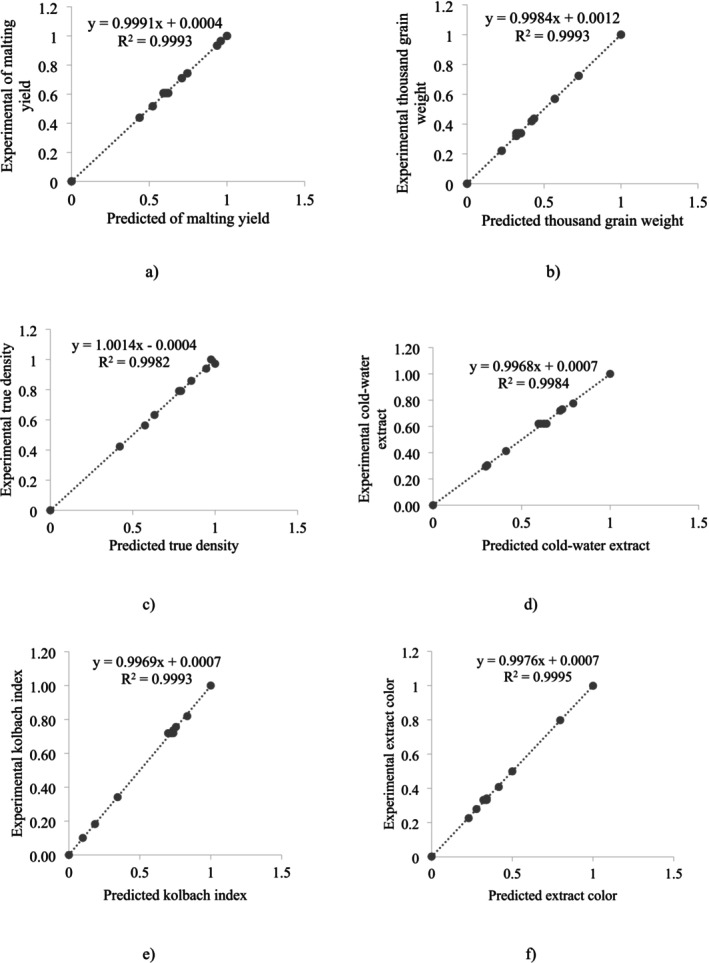
Diagram of predicted changes by the neural network for optimized topology (2‐6‐6) vs. Experimental amount for malting yield (a), thousand grain weight (b), true density (c), cold‐water extract (d), kolbach index (e), and (f) extract color.

The high correlation coefficients presented in Figure 5, which compare the predicted values generated by this optimized network against laboratory data for six output variables, further substantiate the high accuracy of the model. It has been established that the selected models exhibit a remarkable precision in predicting the experimental data (*R* ^2^ > 0.998). Ajayi et al. (2023) also employed ANN modeling to predict and identify the best quality of grains for the malting process; their results showed that this modeling approach is highly effective for improving and predicting the malting process (Ajayi et al. 2023). Additionally, the results from Boniecki et al. (2022) demonstrated the suitability of artificial neural networks in predicting data obtained from the malting process and improving decision‐making efficiency (Boniecki et al. 2022).

### 3.9 The Comparison of the RSM With ANN for Predicting the Characteristics Measured in the Process of Millet Grain Malting

Based on the analysis of Table 2 and Figure 5, it was found that both approaches (RSM and ANN) exhibited high correlation coefficients, making them effective for predicting the properties of millet malt influenced by different steeping and germination periods (Table 4). Nevertheless, the ANN model proved to be more effective than the RSM method in predicting the characteristics of millet malt during the malting process. Similarly, Ghodsvali et al. (2016) showed that ANN is more effective than RSM in predicting the characteristics of barley malt (Ghodsvali et al. 2016).

**TABLE 4:** Comparison of the accuracy of response surface method and ANN in predicting millet malt characteristics under the influence of steeping time and germination.

Prediction method	*R* ^2^ between measured parameters					
	Malting yield	Thousand grain weight	True density	Cold‐water extract	Kolbach index	Extract color
Response surface	0.980	0.936	0.869	0.753	0.969	0.960
Artificial neural network	0.999	0.999	0.998	0.998	0.999	0.999

## 4 Conclusion

In this study, RSM and ANN were utilized to predict the variations in specific characteristics of malt produced from millet, affected by the duration of steeping and germination. The findings reveal that an increase in steeping and germination time leads to a decrease in the traits of the malt grain (malting efficiency, thousand grain weight, and true density), while the properties associated with the extract from millet malt (efficiency of cold‐water extract, Kolbach index, and extract color) improve. The results also showed that, in order to achieve millet malt of desired quality, the steeping time should be 42.54 h, and the germination time should be 9 days. Additionally, it was found that the ANN was more effective in predicting the characteristics of millet malt under different steeping and germination periods. Using these models can increase the speed of predicting the physicochemical properties of millet malt and accelerate the decision‐making process in the malting process.

## Author Contributions

**Fatemeh Erfaniannejad Hosseini Nabadou:** investigation (equal), methodology (equal), writing – original draft (equal). **Masoumeh Moghimi:** project administration (equal), writing – review and editing (equal). **Aminallah Tahmasebi:** methodology (equal), software (equal). **Hamid Bakhshabadi:** software (equal), writing – review and editing (equal).

## Conflicts of Interest

The authors declare no conflicts of interest.

## Data Availability

The authors confirm that the data supporting the findings of this study are available within the article.
